# The multi-omics and Mendelian randomization analyses unveiled potential marker genes in the progression of glioblastoma

**DOI:** 10.1097/MD.0000000000046634

**Published:** 2026-01-23

**Authors:** Jin Hai, Chu Guangxin, Du Bang, Shen Hao, Zhang Xu, Ligang Chen

**Affiliations:** a Department of Neurosurgery, The General Hospital of Northern Theater Command, Shenyang, Liaoning, China; b Bengbu Medical University, Bengbu, Anhui, China.

**Keywords:** alternative splicing, glioma, machine learning, Mendelian randomization, prognostic mode

## Abstract

In glioma research, identifying key molecules for predicting patient prognosis is challenging due to high heterogeneity. This study explores the correlation between alternative splicing (AS) and glioma prognosis, aiming to identify molecular markers for potential treatments. Retrieve transcriptomic and clinical data of glioma samples from the cancer genome atlas, eQTLGen database, and GWAS Catalog database. Build a predictive model for patient prognosis using the lasso-cox method. Machine learning is employed to identify the most diagnostically and predictively valuable biomarkers or features. Mendelian randomization analysis is employed to evaluate the causal relationship between genes and diseases. gene ontology, Kyoto encyclopedia of genes and genomes are utilized to explore the potential pathways and mechanisms of genes. The lasso algorithm was employed to screen various AS-related genes across different types, resulting in distinct gene sets. Subsequently, predictive models for patient prognosis were constructed. Key genes, including “STEAP3,” “MLC1,” “ARHGAP19,” “LFNG,” “NRG1” and “KLC1” were identified through lasso screening. Mendelian randomization analysis highlighted the significance of NRG1 in glioma development. gene ontology analysis, encompassing biological processes, cellular components, and molecular functions, revealed enrichment in processes such as modulation of chemical synaptic transmission and ion channel activity. Moreover, Kyoto encyclopedia of genes and genomes pathways, such as Calcium signaling, MAPK signaling, cAMP signaling, and Rap1 signaling, were significantly enriched. In conclusion, our model, based on AS genes, effectively predicts glioma prognosis, identifying NRG1 as a significant marker.

## 1. Introduction

Glioblastoma is a common primary intracranial tumor that originates from brain glial cells.^[1]^ Although the incidence and mortality of brain tumors are increasing year by year, there are significant differences in different regions, possibly influenced by various complex factors such as local medical levels, environmental factors, and genetic backgrounds.^[2]^ There are diverse methods for treating glioblastoma, including but not limited to surgical treatment, radiotherapy, chemotherapy, targeted therapy, and immunotherapy.^[3]^ With the rapid development of next-generation sequencing technology, the formulation of treatment plans increasingly relies on in-depth tumor classification, the overall health status of patients, and the personalized needs of patients. This technological advancement enables doctors to more accurately identify genetic mutations and biological characteristics of tumors, tailoring the most suitable treatment plans for patients. However, glioblastoma tissue is highly heterogeneous, leading to significant individual differences in treatment outcomes.^[4]^ Therefore, there is an urgent need for new biomarkers and prognostic models to achieve more precise treatment management for individuals. In-depth research into the molecular mechanisms and biological characteristics of glioblastoma will contribute to the discovery of more effective treatment strategies and provide more personalized treatment plans for patients.

Alternative splicing refers to the phenomenon where a single gene’s same transcript produces multiple different RNA molecules through different selective splicing, resulting in the translation of different proteins.^[5]^ In recent years, research has found a close connection between alternative splicing and human diseases, especially in the field of tumors.^[6]^ In breast cancer research, alternative splicing plays a crucial role in cancer drug resistance and shows potential as a target for cancer treatment.^[7]^ In prostate cancer, the disruption of selective splicing is closely associated with the transcriptional programs activated by androgen receptor, ERG, FOXA1, and MYC, with FOXA1 playing a significant role in primary and metastatic prostate cancer.^[8]^ Additionally, research on circur1 suggests that it can suppress the metastasis of gastric cancer by interacting with heterogeneous nuclear ribonucleoprotein M to regulate the selective splicing of genes involved in the cell migration process.^[9]^ However, current research on the relationship between alternative splicing and gliomas is still relatively limited, mainly confined to in-depth studies of individual molecules. In the future, there is a need to further expand research on multiple alternative splicing-related genes and gliomas to gain a deeper understanding of their potential connections and mechanisms of action.

Mendelian randomization is a statistical method based on Mendelian genetics principles, primarily used to study the causal relationship between genotype and phenotype. In research, the genotype variable of interest is typically treated as the exposure factor, and the phenotype variable as the outcome. Causal effects are assessed by comparing phenotype differences between different genotype groups. This method has many advantages, such as effectively controlling confounding factors, reducing experimental errors, and enhancing the reliability and repeatability of experimental results.^[10,11]^ The value of Mendelian randomization lies in its ability to help establish causal relationships, providing more reliable guidance for policy formulation and clinical practice. Mendelian randomization-eQTL combines the principles of Mendelian randomization with expression quantitative trait loci (eQTL) analysis, utilizing Mendelian randomization to delve into the causal relationship between genotype and gene expression levels based on eQTL results.^[12,13]^ Currently, conventional transcriptome and Mendelian Randomization (MR) analysis are typically used in Mendelian randomization analysis of genes and outcomes. However, there is limited research on MR analysis using single-cell transcriptomes and eQTL. Future studies can further expand the use of these novel technologies to gain a more comprehensive and in-depth understanding of the causal relationship between genotype and disease.

This study employed various types of alternative splicing-related genes to construct a prognostic model for gliomas. The association between alternative splicing-related genes and gliomas was further verified through multi-omics analysis and Mendelian Randomization (MR) analysis.

## 2. Methods and materials

### 2.1. Ethics statement

Ethical approval was not required for this study because all datasets were obtained from a publicly accessible Database.

### 2.2. Data acquisition

Downloaded transcriptome and clinical data for gliomas from the TCGA database, including 516 cancer samples. Clinical data includes survival status, survival time, etc. Downloaded exposure and outcome-related datasets from the eQTLGen database and GWAS Catalog (GCST90241260) database, and conducted MR analysis after organizing the data.

### 2.3. Modeling construction and validation

We constructed a riskScore model using the selected key genes to calculate the riskScore for each glioma patient. To comprehensively assess the impact of these key molecules on patient prognosis, we used cox regression analysis to build a prognostic model. The riskScore for each glioma patient was calculated using the formula: riskScore = [Expression level of Gene 1 × coefficient] + [Expression level of Gene 2 × coefficient] +… + [Expression level of Gene n × coefficient]. Firstly, we used Kaplan–Meier analysis to assess whether there was a significant difference in survival rates between the high-risk and low-risk groups of glioma patients. Next, we plotted the risk survival curve to visually display the survival and death situations of patients in the high and low-risk groups, as well as the differences in key genes between the 2 groups. Finally, by drawing the ROC curve, we evaluated the performance of this predictive model.

### 2.4. Machine learning

Least absolute shrinkage and selection operator (LASSO) algorithm, a widely used feature selection and regularization tool in the fields of statistics and machine learning, is extensively applied to sift through a large number of biomarkers or features for those with high diagnostic and predictive value. In this study, we used patient data to construct a model with the aim of identifying biomarkers of significant value for disease prediction and diagnosis.^[14]^

### 2.5. MR analysis

In exploring the causal relationship between genes and diseases, we employed a rigorous strategy combining meticulous data filtering with various advanced statistical methods. Firstly, from a massive amount of genetic data, we carefully selected single nucleotide polymorphisms (SNPs) significantly associated with specific genes as crucial instrumental variables. The significance level of these SNPs reached the standard of a *P*-value <10^−5^, laying the foundation for our subsequent analysis. To comprehensively assess the complex relationship between genes and diseases, we applied 4 different types of statistical methods, each with its unique advantages and applications. Firstly, the inverse variance weighting method, as one of the classic methods in Mendelian Randomization analysis, precisely calculates the contribution of each gene to the outcome and provides a weighted average based on the reciprocal variance of these contributions, thus offering us a comprehensive and reliable estimation of the results. Secondly, we employed the Weighted Median method, which improves upon inverse variance weighting by conducting a median regression on gene effects and assigning different weights to each gene, effectively enhancing the robustness of estimating causal effects in the presence of impure data. Additionally, we applied the MR-Egger regression method, which fully considers the possibility of introducing impurities and cleverly uses the regression intercept to detect and correct biases introduced by impurities. This method is relatively flexible in impurity detection but may produce larger standard errors when impurities have a significant impact. Finally, we explored the weighted mode algorithm in Mendelian randomization analysis. This method fully considers the diversity and complexity of genetic variation, as well as the potential impact of the heterogeneity of diseases and environmental factors. To ensure the reliability and stability of the results, we conducted thorough validation and sensitivity analysis when applying the weighted mode algorithm.^[10–12]^

### 2.6. Functional enrichment analysis

Functional enrichment analysis, a critical tool in bioinformatics, is utilized to investigate the shared functional characteristics of a group of genes or proteins, revealing their roles in biological processes. The core idea involves comparing the gene set with known biological information to determine whether there is enrichment in specific functional categories or pathways. GO and kyoto encyclopedia of genes and genomes (KEGG) analyses are common methods for functional enrichment analysis. GO analysis is designed to categorize the functions of genes and proteins, displaying their relationships through the construction of a hierarchical graph. GO encompasses 3 main aspects: Biological process, molecular function, and cellular component.^[15]^ The KEGG database includes various metabolic pathways, signaling pathways, and disease pathways for different biological systems. KEGG analysis concentrates more on pathway research, providing a more in-depth understanding of gene function and regulatory networks.^[16]^

### 2.7. Data statistics

Differential analysis between 2 groups was conducted using the Wilcoxon test, and correlation analysis was based on the Spearman correlation test. Survival analysis utilized Kaplan–Meier analysis and log-rank tests. The *P*-values were 2-sided, with *P* < .05 considered statistically significant. R software (version 4.2.2) was used for statistical analysis. Mendelian Randomization analysis required adherence to 3 core assumptions: Instrumental variables are related to the exposure factor: Genetic variations were used as instrumental variables and must be associated with the exposure factor of interest. Instrumental variables are unrelated to confounding factors: Instrumental variables should be unrelated to any confounding factors to ensure the accuracy of the analysis results. Instrumental variables do not affect the outcome unless through the exposure factor: Genetic variations should not directly affect the outcome of interest unless achieved through association with the exposure factor.

## 3. Results

### 3.1. Alternative splicing-related gene screening

**Figure 1.**
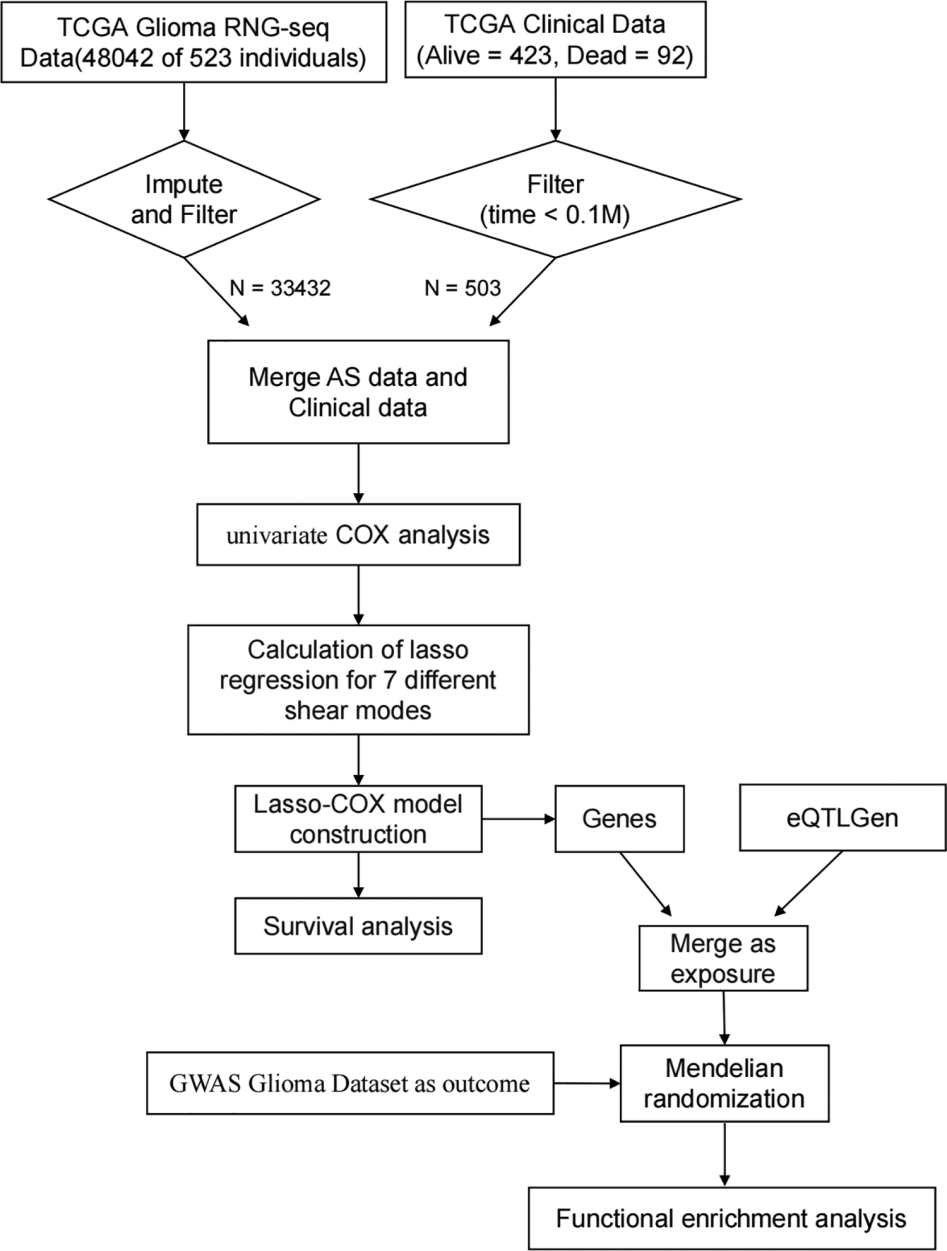
A flowchart of manuscript.

**Figure 2.**
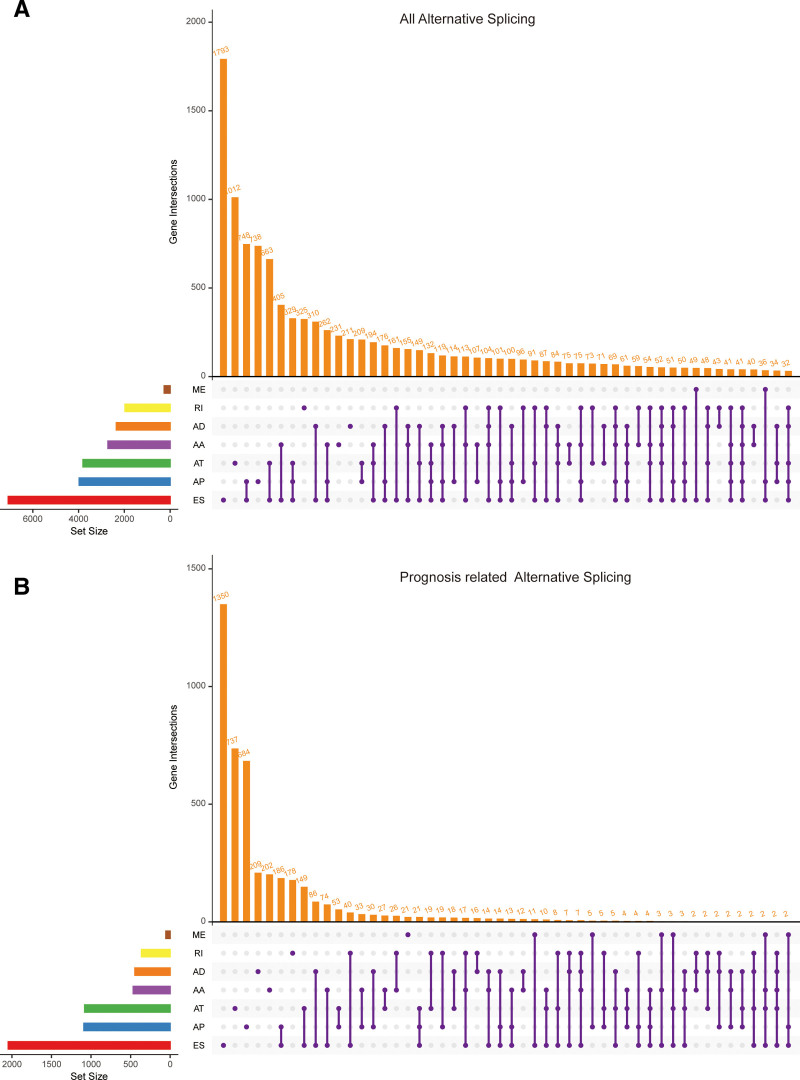
UpSet plot reveals the intersections of different types of alternative splicing-related genes. (A) UpSet plot discovers all alternative splicing-related genes. (B) UpSet plot identifies prognostically relevant alternative splicing-related genes.

**Figure 3.**
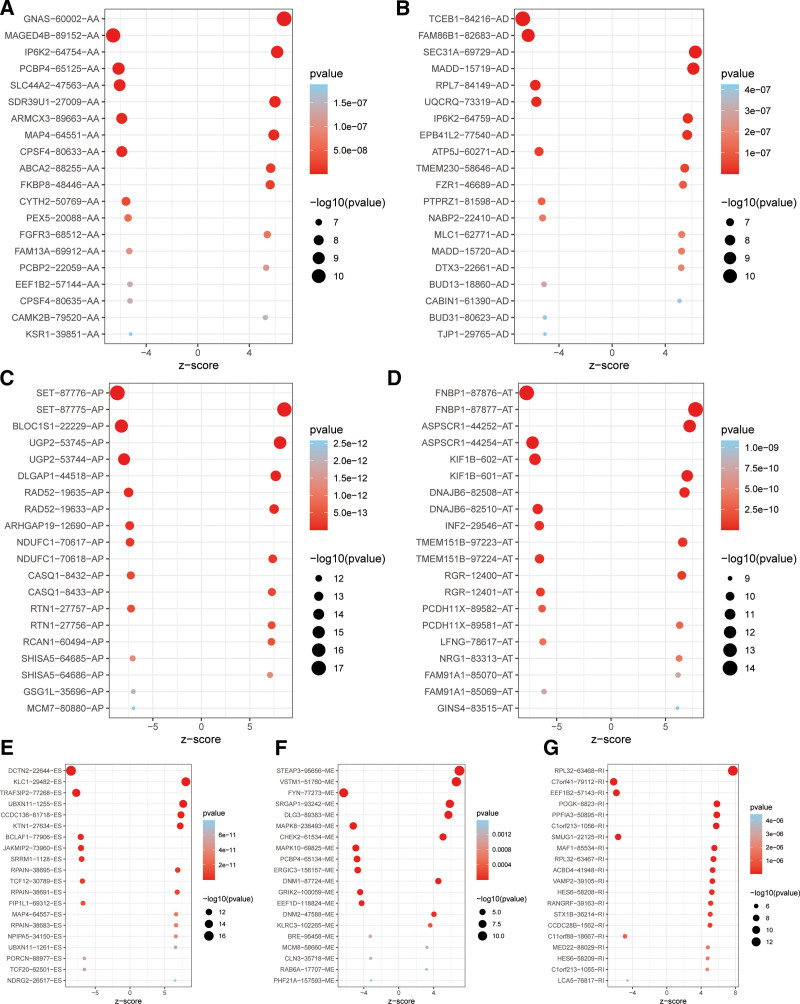
Prognostic analysis displays the top 20 genes in 7 different alternative splicing-related genes.

After understanding the process of alternative splicing and its importance, we designed a study to further explore the underlying mechanisms. To better illustrate this process, we created a flowchart to clearly present the overall framework and steps of the study (Fig. 1). Alternative splicing, also known as selective splicing, is a unique process. In this process, the exons of RNA produced by a major gene or an mRNA precursor transcript can be spliced and reconnected in various ways. This results in the production of mRNA that can be translated into different protein isoforms, allowing a gene to encode multiple proteins.^[5]^ We downloaded data on 7 types of alternatively spliced genes from the MD Anderson Cancer Center’s database. These types include AA (Alternate Acceptor site), AD (Alternate Donor site), AP (Alternate promoter), AT (Alternate terminator), ES (Exon skip), ME (Mutually exclusive exons), and RI (Retained intron). The data shows that there are 231 genes of the AA type, 211 of the AD type, 738 of the AP type, 1012 of the AT type, 1793 of the ES type, and 325 each of ME and RI (Fig. 2A). After obtaining this data, we conducted a prognostic analysis on different types of alternatively spliced genes. During the analysis, some genes were excluded. After the analysis, the number of genes for the AA type decreased to 202, AD to 209, AP to 684, AT to 737, ES to 1350, ME to 21, and RI to 178 (Fig. 2B). Subsequently, we performed additional COX analysis on genes with prognostic value. By calculating the z-score for each gene and filtering based on these values, we identified the top 20 variable splicing-related genes in different types of alternative splicing (Figs. 3A–G).

### 3.2. Lasso-Cox model construction

**Figure 4.**
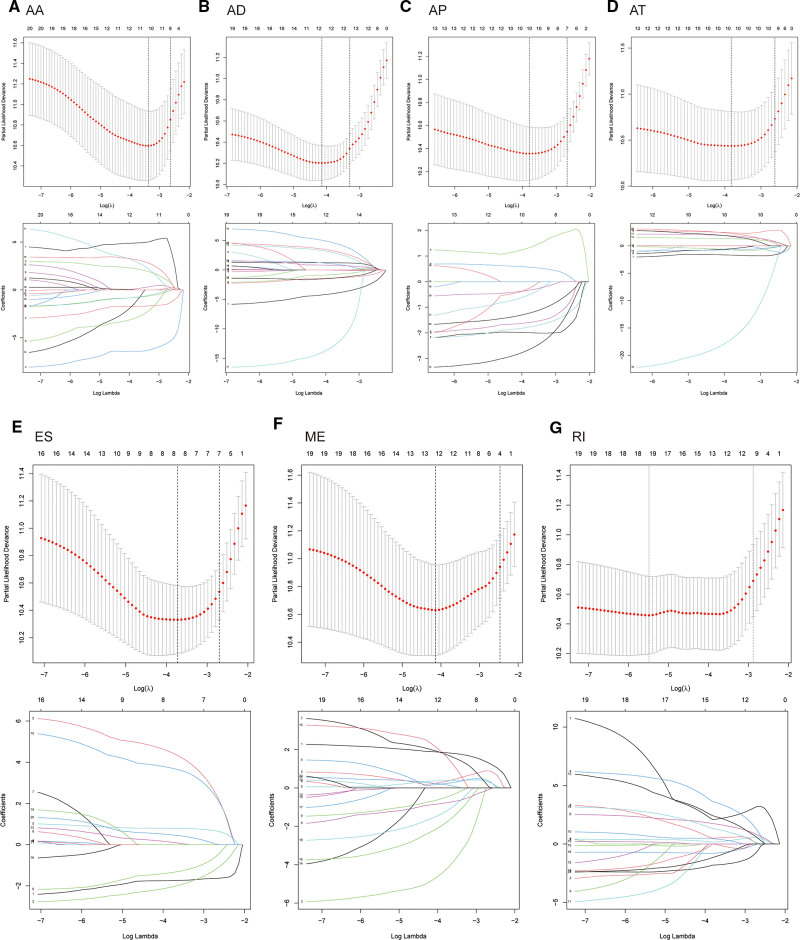
The Lasso-Cox algorithm constructs a prognostic risk model with 7 different alternative splicing-related genes.

Considering that alternatively spliced genes may be closely related to the occurrence and development of glioma patients, we used alternatively spliced genes to construct a prognostic model. First, we performed LASSO analysis to screen the data, in which 11 genes were selected from AA type genes, 12 from AD, 10 from AP, 10 from AT, 8 from ES, 12 from ME, and 14 from RI (Figs. 4A–G). In subsequent analyses, we employed the Cox regression analysis method and, for different datasets, determined the genes used to construct their respective models. The AA gene set was used to construct a model with 6 genes: MAGED4B, IP6K2, PCBP4, FKBP8, FGFR3, and FAM13A. The AD gene set selected 7 genes: TCEB1, FAM86B1, IP6K2, TMEM230, NABP2, MLC1, and BUD13 for model construction. The AP gene set based its model on 6 genes: DLGAP1, RAD52, ARHGAP19, CASQ1, GSG1L, and MCM7. The AT gene set consisted of 9 genes: FNBP1, ASPSCR1, INF2, RGR, PCDH11X, LFNG, NRG1, FAM91A1, and GINS4 for model construction. The ES gene set based its model on 7 genes: DCTN2, KLC1, TRAF3IP2, CCDC136, RPAIN. The AD dataset was selected 7 genes: TCEB1, FAM86B1, IP6K2, TMEM230, NABP2, MLC1, and BUD13 for model construction. The ME gene set based its model on 7 genes: STEAP3, FYN, MAPK8, CHEK2, KLRC3, BRE, and CLN3. The RI gene set used 9 genes: RPL32, C7orf41, POGK, C1orf213, VAMP2, RANGRF, C11orf88, HES6, and LCA5 for model construction. Based on these genes, we calculated the Risk Score for each sample according to the corresponding formula.

### 3.3. Model validation

**Figure 5.**
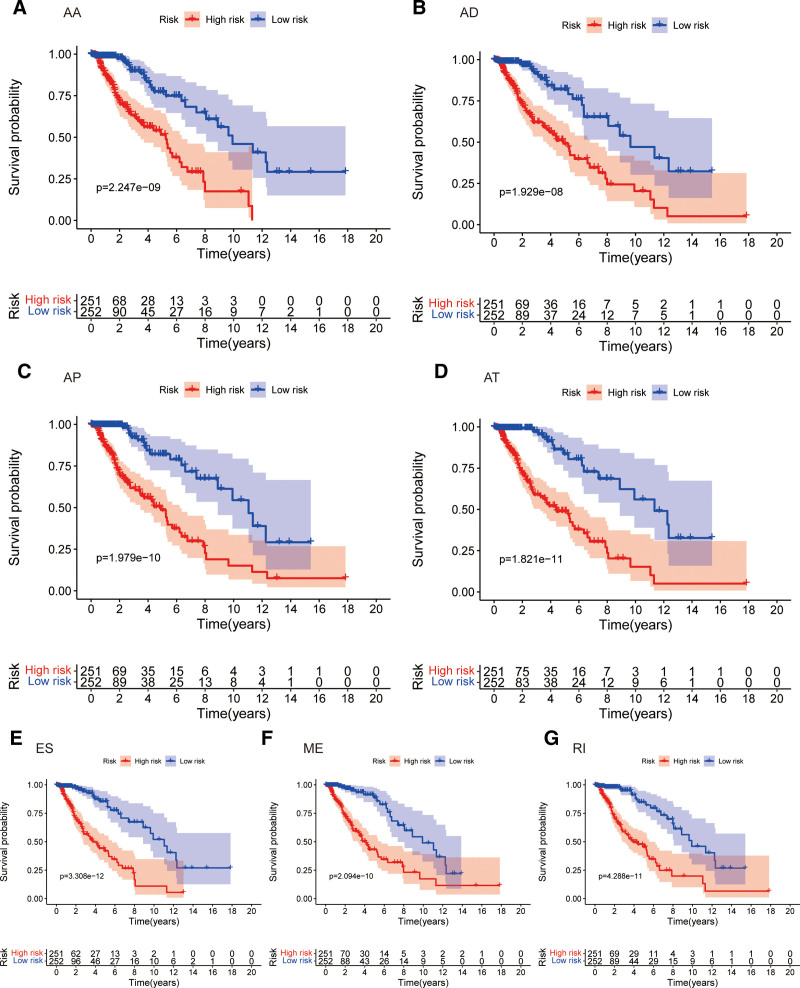
Kaplan–Meier analysis for the riskScore model with 7 different alternative splicing-related genes.

**Figure 6.**
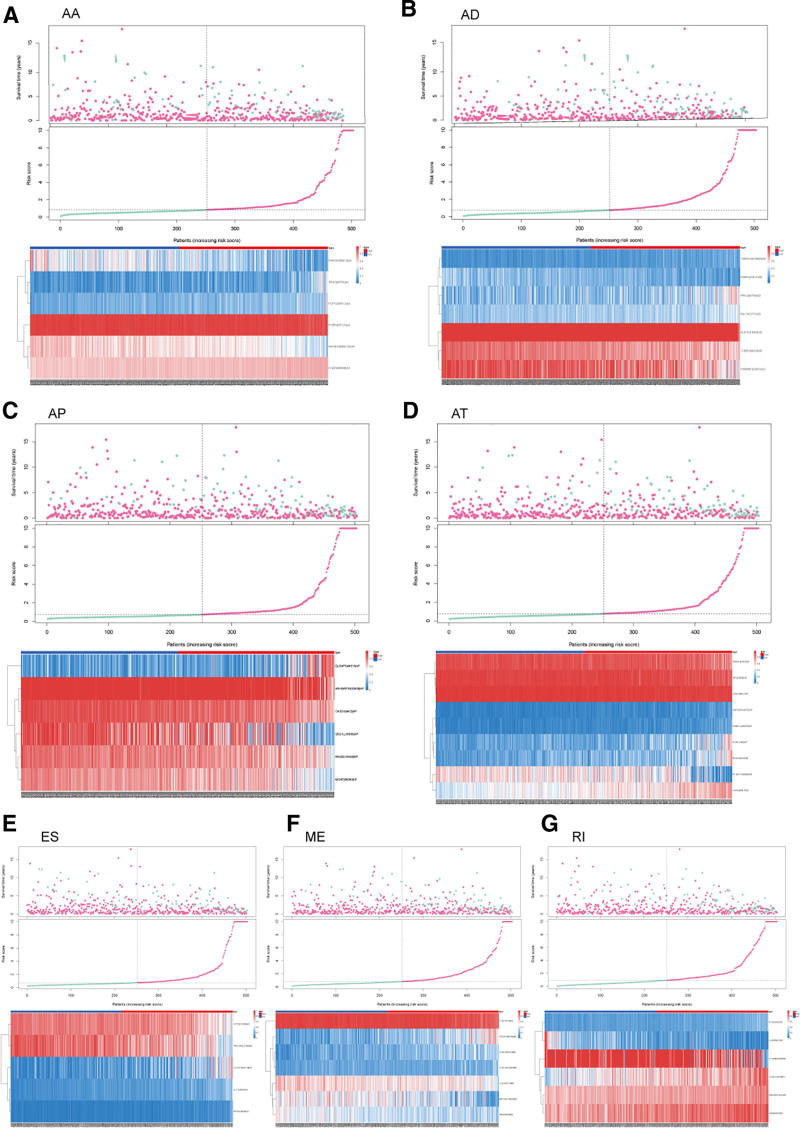
Heatmap and risk curve analysis of the riskScore model with 7 different alternative splicing-related genes.

**Figure 7.**
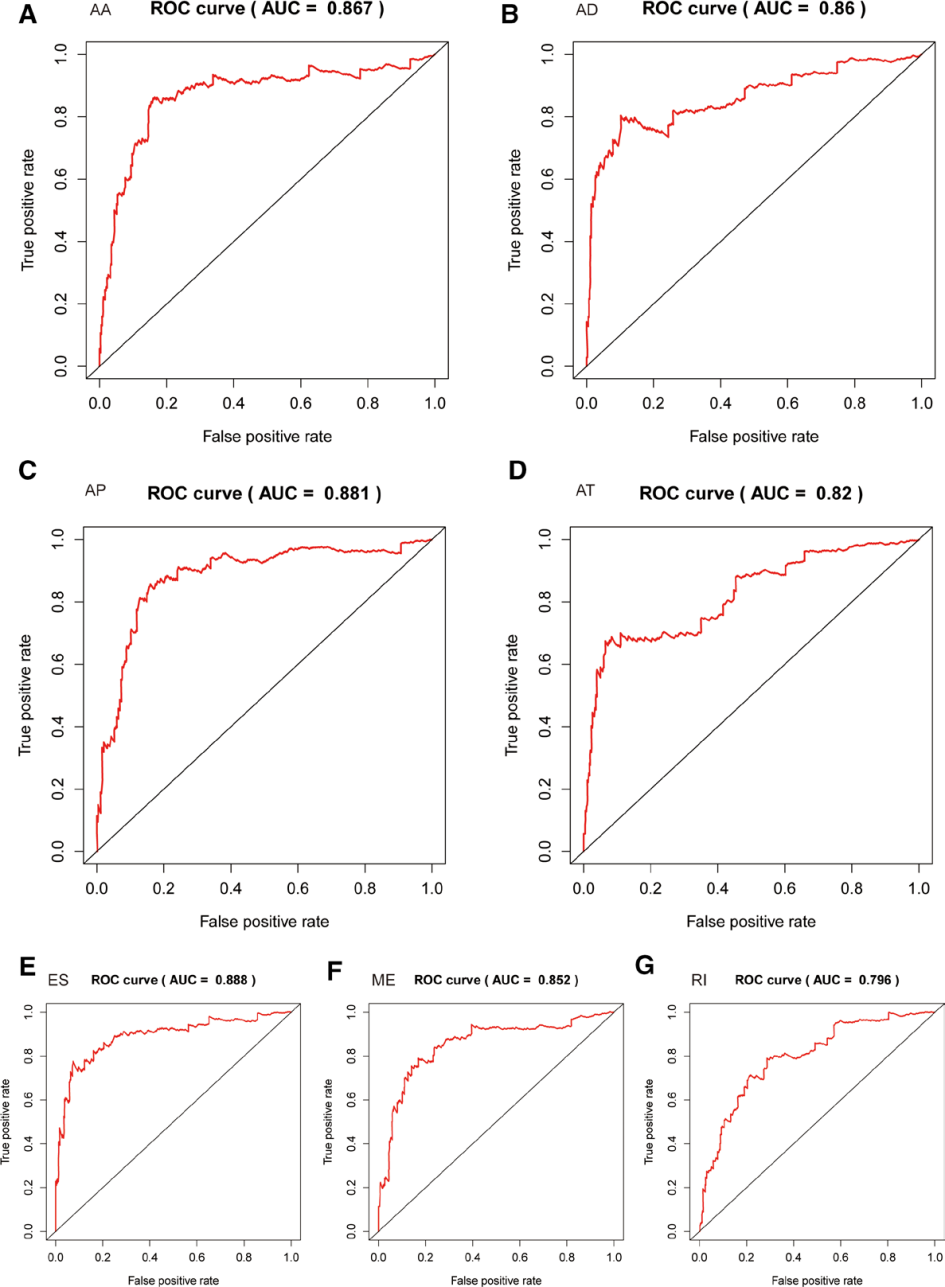
ROC analysis of the riskScore Model with 7 different alternative splicing-related genes.

Considering the correlation between the risk score and patient prognosis, we analyzed the predictive effects of models constructed by different types of alternative splicing. It can be seen that among the 7 models, patients with high-risk scores all had poor prognoses (Figs. 5A–G). To further study the survival status of the 2 groups of patients, we found a higher mortality rate in the high-risk group (Figs. 6A–G). ROC curves showed that the average AUC values of prognosis predictions for the AA-related gene model reached 0.867, AD-related gene model reached 0.860, AP-related gene model reached 0.881, AT-related gene model reached 0.820, ES-related gene model reached 0.888, ME-related gene model reached 0.852, and RI-related gene model reached 0.825 (Figs. 7A–G).

### 3.4. MR analysis

**Figure 8.**
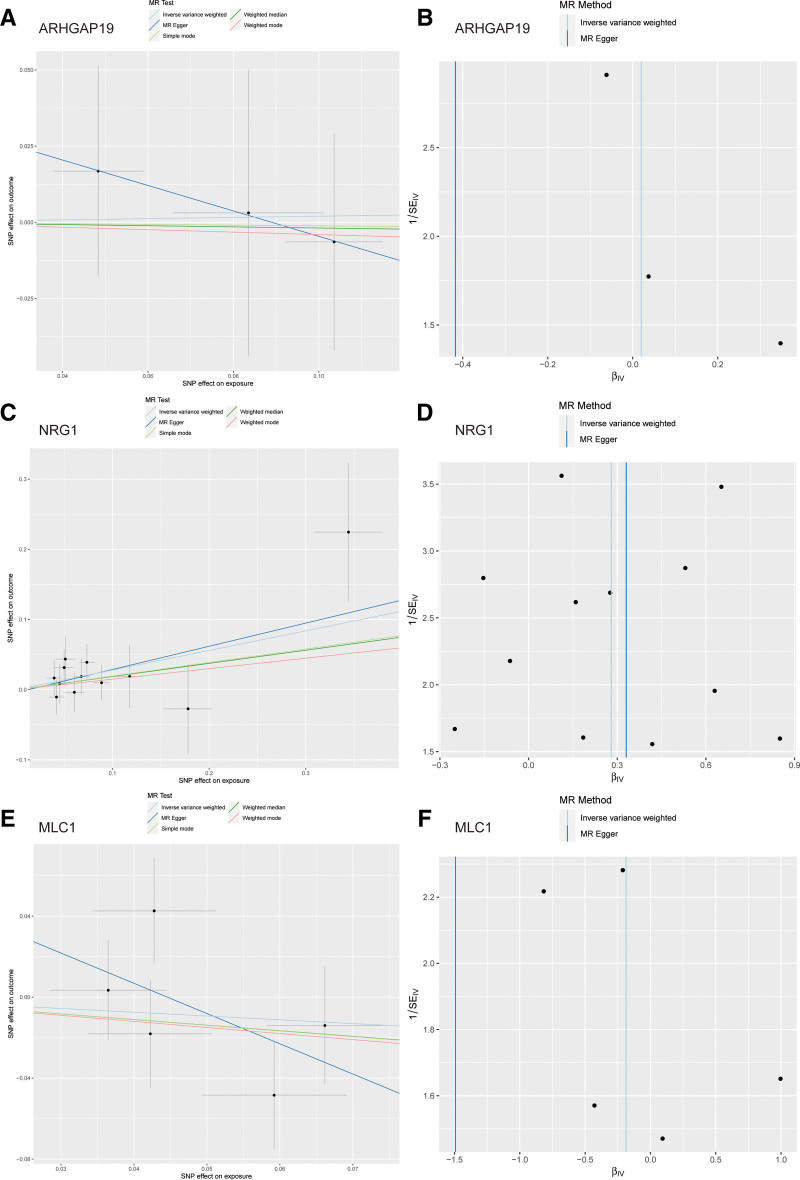
MR analysis reveals the causal relationship between key genes and glioblastoma. (A and B) MR analysis reveals no causal relationship between ARHGAP19 and glioblastoma. (C and D) MR analysis reveals causal relationship between NRG1 and glioblastoma. (E and F) MR analysis reveals no causal relationship between MLC1 and glioblastoma. MR = Mendelian randomization

We employed the Lasso algorithm to further filter critical genes, ultimately confirming 6 genes, namely “STEAP3,” “MLC1,” “ARHGAP19,” “LFNG,” “NRG1” and “KLC” (Fig. S1, Supplemental Digital Content, https://links.lww.com/MD/R93). We obtained the instrumental variables for these genes from the eQTLGen database, setting a screening threshold at a *P*-value of 1 × 10^−5^. Following organization, 5 genes had corresponding SNPs. In subsequent analyses, we chose “STEAP3,” “MLC1,” “ARHGAP19,” “LFNG” and “NRG1” for further investigation. To thoroughly explore the relationship between these genes and gliomas, we selected a glioma-related GWAS dataset conducted Mendelian randomization analysis. The results indicated that the MR analysis results of “NRG1” were statistically significant (*P* < .05), while other genes such as “STEAP3,” “MLC1,” “ARHGAP19” and “LFNG” did not show significant associations (Fig. 8A–F and Fig. S2, Supplemental Digital Content, https://links.lww.com/MD/R93). To ensure the reliability of the analysis results, we also conducted tests for horizontal pleiotropy and leave-one-out sensitivity. The results demonstrated that the *P*-values of “NRG1” were all >.05, indicating no apparent confounding factors.

### 3.5. Functional enrichment analysis

**Figure 9.**
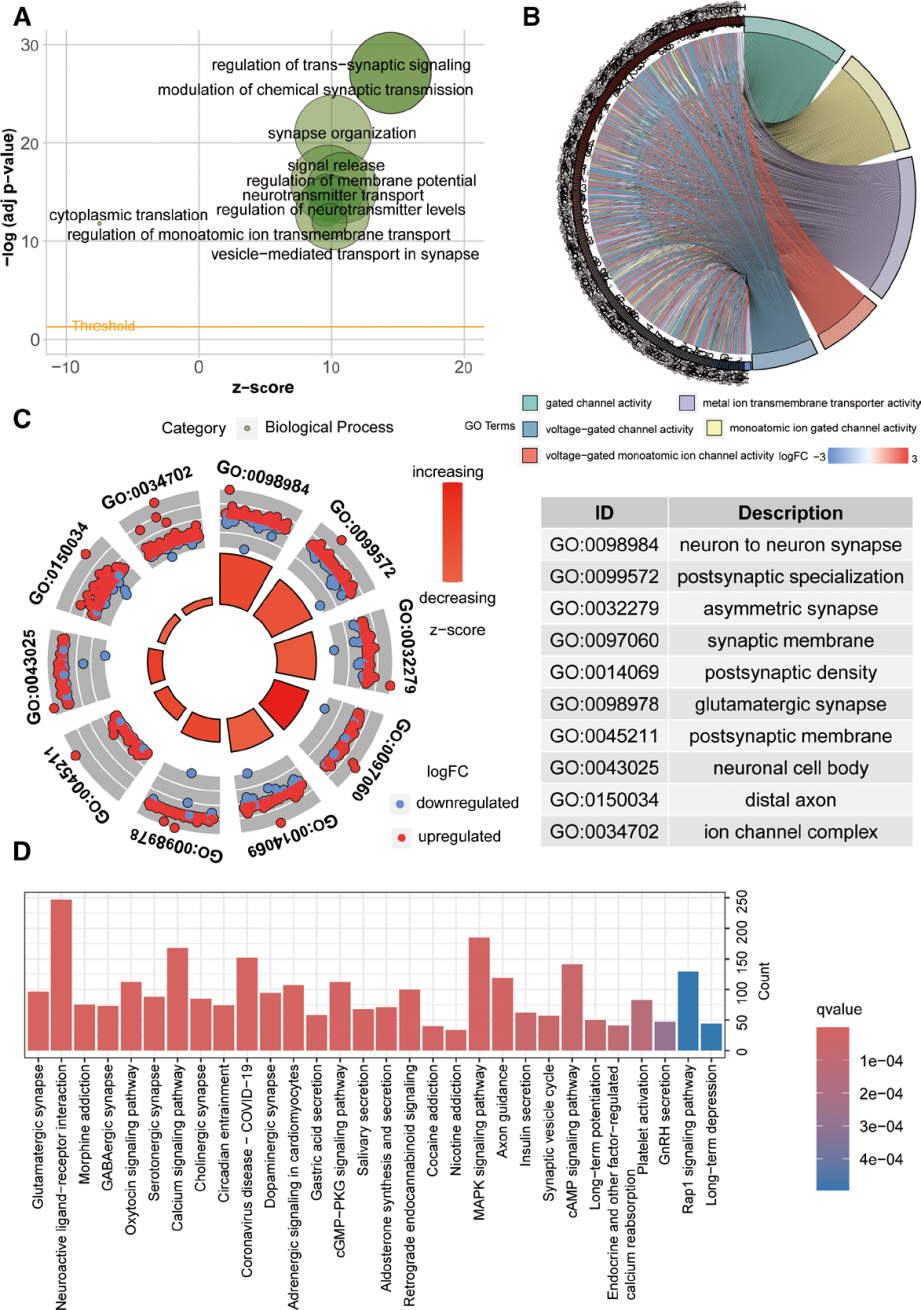
Functional enrichment analysis. (A) BP enrichment analysis of the riskScore model. (B) MF enrichment analysis of the riskScore model. (C) CC enrichment analysis of the riskScore model. (D) KEGG enrichment analysis of the riskScore model. BP = biological process, CC = cellular component, MF = molecular function, KEGG = Kyoto encyclopedia of genes and genomes.

Through Gene Ontology enrichment analysis, we delved into the functional characteristics of genes related to specific diseases, differentially expressed genes under specific conditions, and important functional modules in gene regulatory networks. Biological Process analysis mainly enriched in modulation of chemical synaptic transmission, cytoplasmic translation, regulation of membrane potential (Fig. 9A). In terms of Molecular Function, enrichment mainly focused on gated channel activity, metal ion transmembrane transporter activity (Fig. 9B). Cellular Component analysis mainly enriched in neuron-to-neuron synapse, ion channel complex (Fig. 9C). Subsequently, we further explored the Calcium signaling pathway, MAPK signaling pathway, cAMP signaling pathway, and Rap1 signaling pathway through KEGG enrichment analysis (Fig. 9D).

## 4. Discussion

Glioblastoma is a common primary intracranial tumor, and its formation and development are related to various factors. Prolonged exposure to electromagnetic radiation or X-ray irradiation, unhealthy lifestyles, and psychological factors may increase the risk of glioblastoma. Additionally, genetics is one of the important factors in the formation of glioblastoma.^[1]^ Therefore, it is meaningful to identify molecular markers that are significant for glioblastoma. To better understand the molecular mechanisms of glioblastoma and identify potential therapeutic targets, we conducted prognosis analysis. We identified some key genes associated with the occurrence and development of glioblastoma in this analysis. These genes may play crucial roles in the pathogenesis of glioblastoma, including processes such as cell proliferation, differentiation, migration, and apoptosis. Through in-depth study of these genes, we can further understand the biological characteristics of glioblastoma and potential therapeutic strategies. Additionally, due to the various subtypes and molecular subgroups of glioblastoma, diagnosis and treatment are more challenging. Therefore, we need more precise methods to predict the prognosis of different patients and formulate personalized treatment plans. To achieve this, we further constructed a prognosis model based on hub genes, predicting the prognosis of patients by analyzing their gene expression data. This prognosis model can provide valuable reference information for doctors, assisting in the formulation of more accurate treatment strategies. In summary, through in-depth research into the molecular mechanisms of glioblastoma and the identification of meaningful molecular markers, we can better understand the nature and development trends of this disease. Meanwhile, constructing a prognosis model based on hub genes can provide clinicians with more accurate treatment plans, thereby improving patient survival rates and enhancing their quality of life. These research results provide valuable clues and directions for further exploration of the etiology, pathogenesis, and treatment methods of glioblastoma.

Given the potential association of alternative splicing-related genes with glioblastoma, we conducted an in-depth investigation into their prognostic significance. We identified alternative splicing genes with significant prognostic value through survival analysis. These genes might be pivotal in the development and progression of glioblastoma. For a more precise prediction of patient prognosis, we constructed a Lasso-Cox model using these AS-related genes. The model can comprehensively consider the expression levels of multiple genes, providing a more comprehensive assessment of patient prognosis. Subsequently, we validated the Lasso-Cox model by comparing the prognosis of patients in the high-risk and low-risk groups, finding a markedly worse prognosis in the high-risk group. This indicates that our model can effectively identify patients with an adverse prognosis. For a more in-depth assessment of the model’s predictive efficacy, we employed ROC curves for validation. ROC curves are a widely used tool for evaluating predictive models, with the area under the curve (AUC) calculated to assess accuracy, sensitivity, and specificity.^[17]^ The model exhibited high AUC values, suggesting a favorable predictive performance.

With the cross-fusion of biomedical science and computer science, the application of machine learning technology in the field of medicine is becoming increasingly widespread. This technology brings unprecedented possibilities to medical diagnosis, treatment, prediction, and research. By analyzing patients’ genetic information, lifestyles, and clinical data, machine learning algorithms can predict the risk of patients developing certain diseases in the future, helping doctors take preventive measures in advance and reduce the risk of disease occurrence. Lasso algorithm is a statistical method widely used in regression analysis and feature selection, and it is also one of the commonly used algorithms in machine learning.^[14]^ In this paper, we use the lasso algorithm to further select the genes previously screened and find that constructing a prediction model using genes such as “STEAP3,” “MLC1,” “ARHGAP19,” “LFNG,” “NRG1” and “KLC1” is more valuable. By consulting relevant literature, we found that these genes play important roles in the occurrence and development of glioblastoma. The STEAP3 gene is a prognostic biomarker that can promote glioblastoma progression by regulating the immune microenvironment and the PI3K-AKT pathway.^[18]^ The MLC1 gene promotes the invasion of glioblastoma stem cells in the brain microenvironment.^[19]^ In addition, pan-cancer analysis reveals the roles of LFNG, MFNG, and RFNG genes in tumor prognosis and the microenvironment.^[20]^ The ARHGAP19, NRG1, and KLC1 genes also play certain roles in the development of cancer.^[21–23]^

Mendelian randomization is an analytical method based on the principles of Mendelian genetics, which states that genes are inherited to offspring in a specific manner. In the analysis of Mendelian randomization, we leverage the correlation between genetic markers and phenotypic features to deduce the influence of genetic factors on phenotypic traits.^[11]^ Through MR analysis, we studied the previously screened alternative splicing-related genes, and the results suggested that NRG1 is the sole gene causally associated with glioblastoma. This discovery aligns with prior studies, suggesting that MiR-125a-3p influences the apoptosis and invasion of glioblastoma by regulating Nrg1(23). Members of the neuroregulin protein family also play a vital role in predicting glioblastoma prognosis and conducting immune infiltration analysis.^[24]^

To delve into the distinctions between high and low-risk groups, we employed GO analysis and KEGG analysis, unveiling potential pathways of mechanistic significance. In the realm of biological processes, we noted that the prominently enriched pathways involve the modulation of chemical synaptic transmission and the regulation of membrane potential. Notably, previous studies have indicated a close association between the electric field targeting cell membrane potential and the growth inhibition of HeLa cancer cells.^[25]^ At the molecular functions level, we observed that the enriched pathways include gated channel activity and metal ion transmembrane transporter activity. Past research has indicated the pivotal role of ions like Ca2+, H+, and K + in mitochondrial function, and their significant dysregulation in cancer has been extensively validated.^[26,27]^ In cellular components analysis, the primary enrichment is observed in neuron-to-neuron synapse and ion channel complex. Early research unveiled distortions in the normal spatiotemporal distribution of Ca2 + caused by mutations, ion channel gating, or changes in expression levels. This further prompts the deregulation of downstream substances sensitive to Ca2+, thereby fostering the pathological characteristics of cancer, such as enhanced survival, proliferation, and invasion.^[28,29]^ Remarkably, the principal pathways enriched in KEGG encompass the Calcium signaling pathway, MAPK signaling pathway, cAMP signaling pathway, and Rap1 signaling pathway. Relevant research has indicated that TRIM22 promotes the proliferation of glioblastoma cells by activating MAPK signaling and promoting the degradation of Raf-1.^[30]^ Several Rap1 subtypes have also been confirmed to promote the proliferation and invasion of GBM cells.^[31]^ The Calcium signaling pathway and cAMP signaling pathway are also closely linked to the occurrence and development of tumors.^[32,33]^ These research findings reaffirm our study, underscoring the pivotal role of these signaling pathways in the occurrence and development of tumors.

There are certain limitations in our study that need consideration. Firstly, to ensure the comprehensiveness and accuracy of the study, we extensively explored various public databases. However, some of these databases lack detailed clinical information, potentially introducing certain omissions. To precisely unveil the nature of the disease and prognostic factors, we intend to proactively collect samples and data from our hospital in future research. This will involve more profound exploration and analysis to address this limitation. Secondly, we employed a retrospective research approach, which aids in extracting valuable insights from existing data. Yet, for a more comprehensive evaluation of the predictive performance and practical applicability of the model, prospective studies are essential. Through prospective studies, we can track patients’ conditions and prognoses in real-time, providing a more accurate validation and optimization of the model. Lastly, our prognostic model involves the expression levels of specific genes, but a thorough exploration of how these genes impact the disease process and prognosis mechanisms has not been deeply investigated. In the future, we plan to delve into the inherent connections between these genes and the disease process, offering new perspectives and potential targets for disease treatment and drug development.

In summary, this study holds crucial clinical application value. We conducted a collaborative study using multi-omics analysis combined with MR. The risk score constructed by LASSO-COX has been demonstrated as a reliable and independent biomarker, suitable for predicting the prognosis of glioma patients. Subsequent MR analysis aids in validating the accuracy of hub genes, and functional enrichment analysis helps us delve into mechanisms and perform downstream analysis.

## 5. Conclusion

In summary, we have developed a model for predicting the prognosis of gliomas through the research on key genes involved in alternative splicing. Simultaneously, combining machine learning and Mendelian randomization analysis, we discovered that NRG1 is a biologically significant molecular marker.

## Author contributions

**Data curation:** Chu Guangxin.

**Investigation:** Shen Hao.

**Methodology:** Chu Guangxin.

**Supervision:** Du Bang, Ligang Chen.

**Validation:** Jin Hai, Zhang Xu, Ligang Chen.

**Writing – original draft:** Jin Hai, Du Bang, Shen Hao, Zhang Xu, Ligang Chen.

**Writing – review & editing:** Jin Hai, Ligang Chen.

## Supplementary Material



## References

[1] Ostrom QT Bauchet L Davis FG. The epidemiology of glioma in adults: a “state of the science” review. Neuro-Oncology. 2014;16:896–913. 24842956 10.1093/neuonc/nou087 PMC4057143

[2] Bielecka J Markiewicz-Żukowska R. The influence of nutritional and lifestyle factors on glioma incidence. Nutrients. 2020;12:1812. 32560519 10.3390/nu12061812 PMC7353193

[3] Xu S Tang L Li X Fan F Liu Z. Immunotherapy for glioma: current management and future application. Cancer Lett. 2020;476:1–12. 32044356 10.1016/j.canlet.2020.02.002

[4] Gusyatiner O Hegi ME. Glioma epigenetics: from subclassification to novel treatment options. Semin Cancer Biol. 2018;51:50–8. 29170066 10.1016/j.semcancer.2017.11.010

[5] Wright CJ Smith CWJ Jiggins CD. Alternative splicing as a source of phenotypic diversity. Nat Rev Genet. 2022;23:697–710. 35821097 10.1038/s41576-022-00514-4

[6] Roy Burman D Das S Das C Bhattacharya R. Alternative splicing modulates cancer aggressiveness: role in EMT/metastasis and chemoresistance. Mol Biol Rep. 2021;48:897–914. 33400075 10.1007/s11033-020-06094-y

[7] Deng L Liao L Zhang Y-L. MYC-driven U2SURP regulates alternative splicing of SAT1 to promote triple-negative breast cancer progression. Cancer Lett. 2023;560:216124. 36907504 10.1016/j.canlet.2023.216124

[8] Del Giudice M Foster JG Peirone S. FOXA1 regulates alternative splicing in prostate cancer. Cell Reports. 2022;40:111404. 36170835 10.1016/j.celrep.2022.111404 PMC9532847

[9] Wang X Li J Bian X. CircURI1 interacts with hnRNPM to inhibit metastasis by modulating alternative splicing in gastric cancer. Proc Natl Acad Sci USA. 2021;118:e2012881118. 34385309 10.1073/pnas.2012881118 PMC8379983

[10] Zeitoun T El-Sohemy A. Using mendelian randomization to study the role of iron in health and disease. Int J Mol Sci. 2023;24:13458. 37686261 10.3390/ijms241713458 PMC10487635

[11] Sekula P Del Greco M F Pattaro C Köttgen A. Mendelian randomization as an approach to assess causality using observational data. J Am Soc Nephrol. 2016;27:3253–65. 27486138 10.1681/ASN.2016010098 PMC5084898

[12] Kintu C Soremekun O Kamiza AB. The causal effects of lipid traits on kidney function in Africans: bidirectional and multivariable Mendelian-randomization study. eBioMedicine. 2023;90:104537. 37001235 10.1016/j.ebiom.2023.104537 PMC10070509

[13] Maimaiti A Turhon M Abulaiti A. DNA methylation regulator-mediated modification patterns and risk of intracranial aneurysm: a multi-omics and epigenome-wide association study integrating machine learning, Mendelian randomization, eQTL and mQTL data. J Transl Med. 2023;21:660. 37742034 10.1186/s12967-023-04512-w PMC10518114

[14] Tang G Qi L Sun Z. Evaluation and analysis of incidence and risk factors of lower extremity venous thrombosis after urologic surgeries: a prospective two-center cohort study using LASSO-logistic regression. Int J Surg. 2021;89:105948. 33892158 10.1016/j.ijsu.2021.105948

[15] Shen J Gao H Li B Huang Y Shi Y. The integration of machine learning and multi-omics analysis provides a powerful approach to screen aging-related genes and predict prognosis and immunotherapy efficacy in hepatocellular carcinoma. Aging (Albany NY). 2023;15:6848–64. 37517087 10.18632/aging.204876 PMC10415564

[16] Shen J Sun W Liu J Li J Li Y Gao Y. Metabolism-related signatures is correlated with poor prognosis and immune infiltration in hepatocellular carcinoma via multi-omics analysis and basic experiments. Front Oncol. 2023;13:1130094. 36860325 10.3389/fonc.2023.1130094 PMC9969091

[17] Mandrekar JN. Receiver operating characteristic curve in diagnostic test assessment. J Thorac Oncol. 2010;5:1315–6. 20736804 10.1097/JTO.0b013e3181ec173d

[18] Song Z Zhao Z Zhu S. STEAP3 is a prognostic biomarker that promotes glioma progression by regulating immune microenvironment and PI3K-AKT pathway. Cancer Biomarkers. 2023;38:505–22. 37980651 10.3233/CBM-230217 PMC12412881

[19] Lattier JM De A Chen Z. Megalencephalic leukoencephalopathy with subcortical cysts 1 (MLC1) promotes glioblastoma cell invasion in the brain microenvironment. Oncogene. 2020;39:7253–64. 33040087 10.1038/s41388-020-01503-9 PMC7736299

[20] Gong X Zheng C Jia H. A pan-cancer analysis revealing the role of LFNG, MFNG and RFNG in tumor prognosis and microenvironment. BMC Cancer. 2023;23:1065. 37932706 10.1186/s12885-023-11545-3 PMC10626706

[21] Vajen B Greiwe L Schaffer V. MicroRNA-192-5p inhibits migration of triple negative breast cancer cells and directly regulates Rho GTPase activating protein 19. Genes Chromosomes Cancer. 2021;60:733–42. 34296808 10.1002/gcc.22982

[22] Moamer A Hachim IY Binothman N Wang N Lebrun J-J Ali S. A role for kinesin-1 subunits KIF5B/KLC1 in regulating epithelial mesenchymal plasticity in breast tumorigenesis. EBioMedicine. 2019;45:92–107. 31204277 10.1016/j.ebiom.2019.06.009 PMC6642081

[23] Yin F Zhang JN Wang SW. MiR-125a-3p regulates glioma apoptosis and invasion by regulating Nrg1. PLoS One. 2015;10:e0116759. 25560389 10.1371/journal.pone.0116759 PMC4283963

[24] Zhao WJ Ou GY Lin WW. Integrative analysis of neuregulin family members-related tumor microenvironment for predicting the prognosis in gliomas. Front Immunol. 2021;12:682415. 34054873 10.3389/fimmu.2021.682415 PMC8155525

[25] Li X Yang F Rubinsky B. A Correlation between electric fields that target the cell membrane potential and dividing HeLa cancer cell growth inhibition. IEEE Trans Biomed Eng. 2021;68:1951–6. 33275576 10.1109/TBME.2020.3042650

[26] Pedersen SF Flinck M Pardo LA. The interplay between dysregulated ion transport and mitochondrial architecture as a dangerous liaison in cancer. Int J Mol Sci. 2021;22:5209. 34069047 10.3390/ijms22105209 PMC8156689

[27] Alvear-Arias JJ Pena-Pichicoi A Carrillo C. Role of voltage-gated proton channel (Hv1) in cancer biology. Front Pharmacol. 2023;14:1175702. 37153807 10.3389/fphar.2023.1175702 PMC10157179

[28] Shapovalov G Ritaine A Skryma R Prevarskaya N. Role of TRP ion channels in cancer and tumorigenesis. Semin Immunopathol. 2016;38:357–69. 26842901 10.1007/s00281-015-0525-1

[29] Anderson KJ Cormier RT Scott PM. Role of ion channels in gastrointestinal cancer. World J Gastroenterol. 2019;25:5732–72. 31636470 10.3748/wjg.v25.i38.5732 PMC6801186

[30] Fei X Dou Y-n Sun K. TRIM22 promotes the proliferation of glioblastoma cells by activating MAPK signaling and accelerating the degradation of Raf-1. Exp Mol Med. 2023;55:1203–17. 37258577 10.1038/s12276-023-01007-y PMC10318069

[31] Manzano S Gutierrez-Uzquiza A Bragado P Cuesta AM Guerrero C Porras A. C3G Protein, a new player in glioblastoma. Int J Mol Sci. 2021;22:10018. 34576182 10.3390/ijms221810018 PMC8466177

[32] Pei Z Lee K-C Khan A Erisnor G Wang H-Y. Pathway analysis of glutamate-mediated, calcium-related signaling in glioma progression. Biochem Pharmacol. 2020;176:113814. 31954716 10.1016/j.bcp.2020.113814 PMC8403340

[33] Qin Z Huang Y Li Z. Glioblastoma vascular plasticity limits effector T-cell infiltration and is blocked by cAMP activation. Cancer Immunol Res. 2023;11:1351–66. 37540804 10.1158/2326-6066.CIR-22-0872

