# Supplementary material for: The multi-omics and Mendelian randomization analyses unveiled potential marker genes in the progression of glioblastoma
Source: Medicine (Baltimore). 2026 Jan 23;105(4):e46634. doi: 10.1097/MD.0000000000046634 (PMC12851673; doi:10.1097/MD.0000000000046634)

Supplementary Figure 1. The Lasso algorithm constructs a model with 6 different key genes

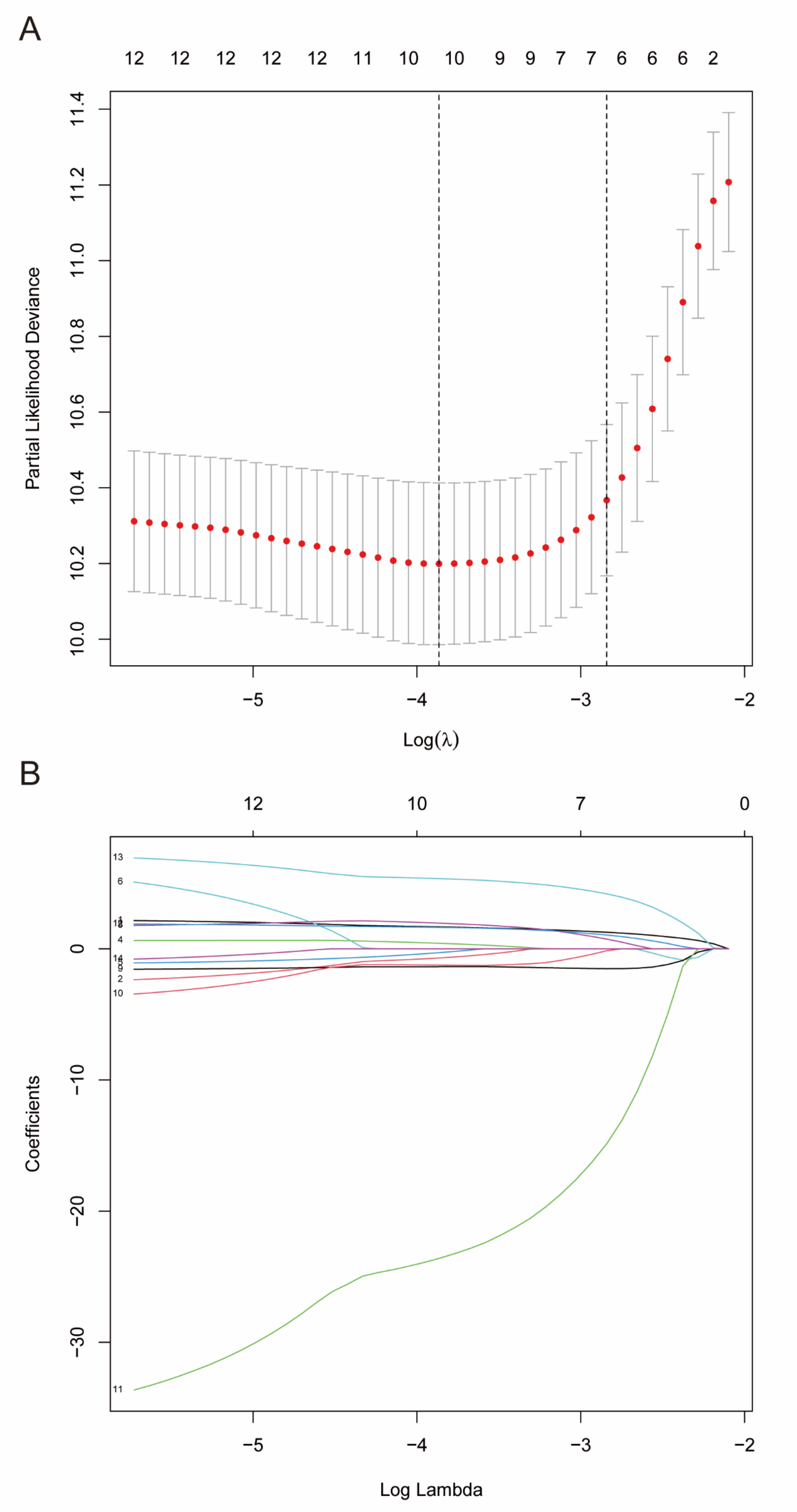

Supplementary Figure 2. MR analysis reveals the causal relationship between key genes and glioblastoma

(A-B) Mendelian randomization analysis reveals no causal relationship between STEAP3 and glioblastoma

(C-D) Mendelian randomization analysis reveals causal relationship between LFNG and glioblastoma

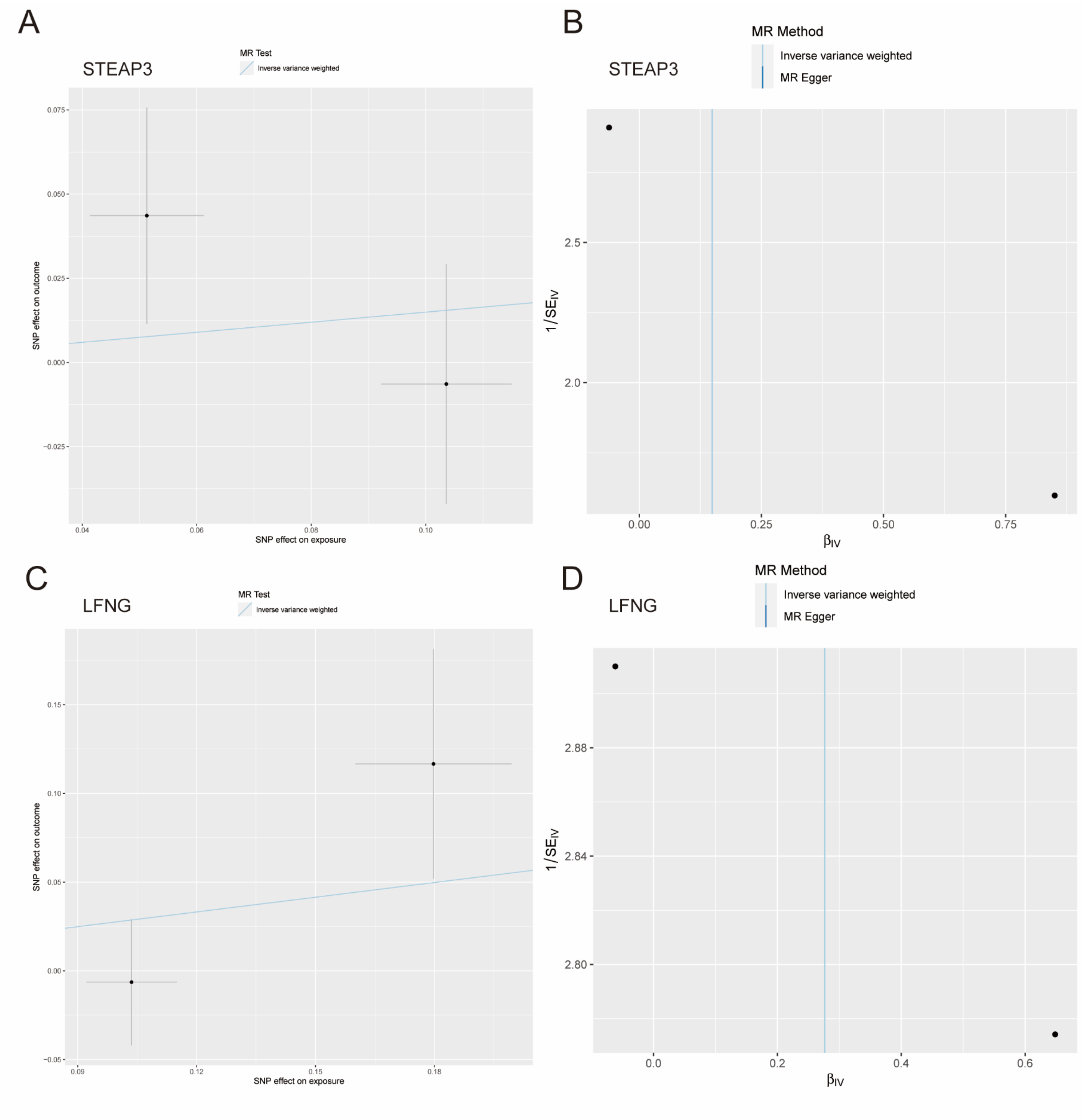

Supplement: Supplementary file 1 [file medi-105-e46634-s001.pdf]
